# EGFR Signaling in Lung Fibrosis

**DOI:** 10.3390/cells11060986

**Published:** 2022-03-14

**Authors:** Fabian Schramm, Liliana Schaefer, Malgorzata Wygrecka

**Affiliations:** 1 Center for Infection and Genomics of the Lung (CIGL), Universities of Giessen and Marburg, 35392 Giessen, Germany; fabian.schramm@med.uni-giessen.de; 2 Institute of Pharmacology and Toxicology, Goethe University Frankfurt, 60590 Frankfurt, Germany; schaefer@med.uni-frankfurt.de

**Keywords:** epidermal growth factor, epidermal growth factor receptor, ErbB-signaling, pulmonary fibrosis, idiopathic pulmonary fibrosis, lung fibrosis, tyrosine kinase inhibitor, TGF-α, TGF-β, amphiregulin, neuregulin 1

## Abstract

In this review article, we will first provide a brief overview of the ErbB receptor–ligand system and its importance in developmental and physiological processes. We will then review the literature regarding the role of ErbB receptors and their ligands in the maladaptive remodeling of lung tissue, with special emphasis on idiopathic pulmonary fibrosis (IPF). Here we will focus on the pathways and cellular processes contributing to epithelial–mesenchymal miscommunication seen in this pathology. We will also provide an overview of the in vivo studies addressing the efficacy of different ErbB signaling inhibitors in experimental models of lung injury and highlight how such studies may contribute to our understanding of ErbB biology in the lung. Finally, we will discuss what we learned from clinical applications of the ErbB1 signaling inhibitors in cancer in order to advance clinical trials in IPF.

## 1. Introduction

The epidermal growth factor (EGF) receptor (EGFR), belongs to the family of the ErbB tyrosine kinase receptors [1,2]. EGFR is also known as ErbB1 or HER1. Other members of this family are ErbB2 (HER2) [3], ErbB3 (HER3) [4] and ErbB4 (HER4) [5]. Following ligand binding, ErbB receptors form homo- or heterodimers that are autophosphorylated on tyrosine residues by intrinsic tyrosine kinase activity and mediate signal transduction to the nucleus [6]. Several ErbB ligands have been identified thus far, including EGF [7,8], transforming growth factor-α (TGF-α) [9], amphiregulin (AREG) [10], heparin-binding EGF-like growth factor (HB-EGF) [11], betacellulin (BC) [12], epiregulin (EREG) [13], epigen (EPG) [14] and neuregulins (NRGs) [15]. All ErbB ligands exist as membrane-anchored precursors that are released in an active form by enzymatic cleavage to the extracellular milieu. Matrix metalloproteinases (MMPs) and A disintegrin and metalloproteinases (ADAMs) were found to be responsible for the shedding of ErbB ligands [16,17,18]. The main sheddases of ErbB ligands are ADAM-10 and -17. ADAM-10 was reported to release EGF and BC [18], whereas ADAM-17 to cleave TGF-α, AREG, HB-EGF and EREG. In addition, MMP-3 was described to release HB-EGF from rat ventral prostate epithelial cells [17].

**Figure 1:**
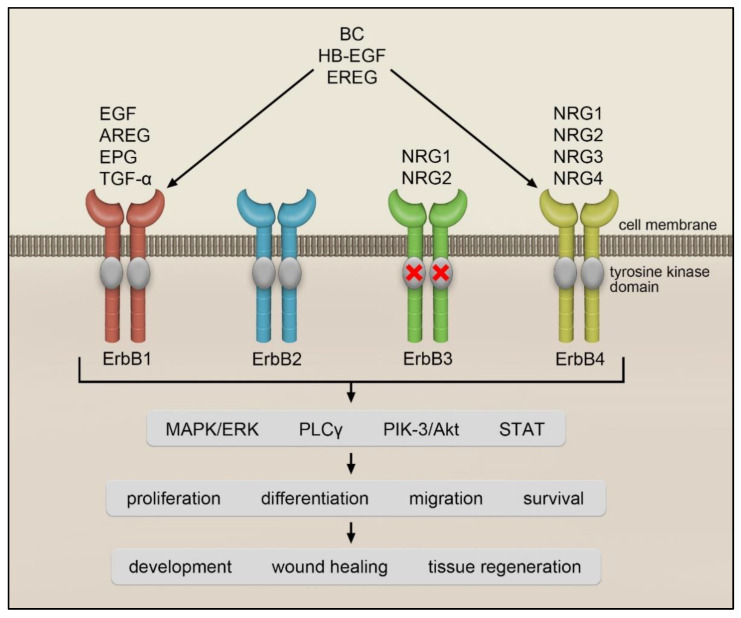
**ErbB receptors and their ligands**. ErbB2 has no known ligand, while ErbB3 lacks intrinsic kinase activity (indicated by a cross). Following ligand binding, the receptors activate several downstream signaling pathways thereby regulating cell growth, proliferation, and survival. These processes play an important role in development, wound healing and tissue regeneration. EGF, epidermal growth factor; TGFα, transforming growth factor-α; EPG, epigen; AREG, amphiregulin; EREG, epiregulin; BC, betacellulin; HB-EGF, heparin-binding EGF-like growth factor; NRG1-4, neuregulin 1-4; STAT, signal transducer and activator of transcription; MAPK/ERK, mitogen-activated protein kinase/extracellular signal-regulated kinase; PIK3/Akt, phosphoinositide 3-kinase/protein kinase B; PLCγ, phospholipase Cγ.

EGF is a prototype member of the ErbB ligand family. This polypeptide was originally identified in the submaxillary glands of mice and the urine of humans. EGF was discovered by Stanley Cohen while working with Rita Levi-Montalcini on nerve growth factors. For these discoveries, Stanley Cohen and Rita Levi-Montalcini were awarded the Nobel Prize in Physiology or Medicine in 1986 [19]. EGF, TGF-α, AREG and EPG only interact with ErbB1, while EREG, BC and HB-EGF bind to ErbB1 and ErbB4. A heterogeneous group of ligands called neuregulins (NRG) interacts with ErbB3 and ErbB4 [15] (Figure 1). The NRG family is composed of NRG1 isoforms, NRG2 (also known as neural- and thymus-derived activator for ErbB kinases (NTAK)) [11,20], NRG3 [21] and NRG4 [22]. The different NRG1 isoforms can be categorized into three smaller groups: type I NRG1, which includes heregulins (HRGs) [23], neu differentiation factor (NDF) [24,25] and acetylcholine receptor-inducing activity (ARIA) [26]; type II NRG1, which contains the glial growth factors (GGFs) [24] and type III NRG1, which comprises the sensory and motor neuron-derived factor (SMDF) [27]. While NRG1 and NRG2 bind to ErbB3 and ErbB4, NRG3 and NRG4 only interact with ErbB4 [15] (see Figure 1).

So far, there is no known ligand for the ErbB2 receptor [28], however, ErbB2 is a preferred partner when forming heterodimers with other ErbB receptors [29]. For instance, binding of EGF to ErbB1 may induce phosphorylation of ErbB2 [30] and thus marked amplification of a signal. Furthermore, binding of NRG1 or NRG2 to ErbB3, which lacks intrinsic catalytic activity, can trigger ErbB3 phosphorylation by the kinase-active partner ErbB2 and thus transduction of a potent mitogenic signal [31]. 

Upon ligand binding, ErbB receptors trigger phosphorylation of diverse effector proteins and activation of multiple downstream signaling pathways. There is a number of excellent reviews describing downstream signaling pathways initiated by ErbB receptors for those interested in this topic [8,15]. Here, only some of the major ErbB downstream signaling pathways are listed. These include: mitogen-activated protein kinase (MAPK)/extracellular signal-regulated kinase (ERK) pathway [32], phosphoinositide 3-kinase (PIK3)/protein kinase B (Akt) pathway [33], phospholipase Cγ (PLCγ) pathway [34], or signal transducers and activators of transcription (STAT) pathway [8]. Activation of the ErbB receptors regulates cell growth, proliferation, and survival and it is associated with a number of biological processes such as organ development, tissue regeneration, and wound healing [35]. The importance of the ErbB-mediated signaling for organogenesis is underscored by the studies showing that inhibition of ErbB1 impairs the development of epithelia in almost every organ including the heart [36], skin [37], lung, brain, kidney and liver [38,39], leading to mouse death shortly after birth [37,39]. Deficiency in single ErbB ligands results in similar pathological changes as the lack of the receptors themselves [40,41]. 

Besides its role in many developmental and physiological processes, overactivation of ErbB signaling has been widely described in many forms of cancer, including glioblastoma [42] and lung [43], breast and ovarian cancer [44,45,46]. These findings led to the development of ErbB signaling inhibitors for the treatment of the aforementioned pathologies [47,48]. Emerging interest in the ErbB signaling and its function during carcinogenesis brought attention to the role of the ErbB receptors and their ligands in other hyperproliferative diseases including lung fibrosis [49].

In this review, we will focus on the ErbB signaling in lung fibrosis, with special emphasis on idiopathic pulmonary fibrosis (IPF). We will discuss the implications of the ErbB signaling in processes that are hallmarks of the maladaptive remodeling of lung tissue. Lastly, we will critically discuss recent advances and future perspectives in targeting the ErbB signaling for lung fibrosis therapy.

## 2. Idiopathic Pulmonary Fibrosis

Idiopathic pulmonary fibrosis (IPF) is one of the most common forms of diffuse parenchymal lung diseases and is characterized by excessive deposition of extracellular matrix proteins in the lung [50]. IPF is an age-related disease, and with the human population aging worldwide, the economic burden of IPF is expected to constantly increase in the future. The pathomechanism of IPF remains elusive, with preferred concepts of disease pathobiology involving recurrent microinjuries to a genetically predisposed alveolar epithelium, followed by an abnormal activation of mesenchymal cells ((myo)fibroblasts), their expansion and massive accumulation of collagens in the lung. Aggregates of active (myo)fibroblasts, so-called fibroblastic foci, are typical histological features of IPF [50]. Fibroblastic foci are often covered by aberrant basaloid cells [51] and MUC5B-producing airway secretory cells [52]. Repopulation of alveoli by abnormal airway epithelial cells is associated with the formation of honeycomb cysts, which are indicators of advanced fibrosis and poor prognosis [50]. Active involvement of airway epithelial cells in the pathogenesis of IPF is reinforced by the fact that the gain-of-function *MUC5B* promoter variant rs35705950 is the dominant risk factor for disease development [53]. Although, it is not entirely clear how increased expression of MUC5B contributes to IPF pathobiology, the study by Hancock et al. [52] linked MUC5B overexpression to impaired mucociliary clearance accompanied by progressive lung tissue scarring.

Increased expression of several cytokine/growth factors have been considered to drive profibrotic processes in the lung, including transforming growth factor-β1 (TGF-β1), platelet-derived growth factor-BB (PDGF-BB), connective tissue growth factor (CTGF), vascular endothelial growth factor (VEGF), and tumor necrosis factor-α (TNF-α). These mediators contribute to lung tissue scarring by deregulating activation, survival, proliferation, and differentiation of a variety of cells, including mesenchymal and epithelial cells [54,55]. For instance, TGF-β1, which is stored in the ECM in a latent form and activated by cell contractile forces, drives the conversion of fibroblasts to matrix-producing myofibroblasts. Excessive deposition of ECM and its stiffening lower the threshold for TGF-β1 activation thereby creating a self-amplifying loop that promotes the expansion of myofibroblasts and fibrosis development [56]. These processes may be enhanced by factors such as interleukin (IL)-6, IL-1β, or TNF-α, which potentiate TGF-β1 expression and activation of the TGF-β1 signaling pathway [57,58]. All these changes are aggravated by the resistance of IPF (myo)fibroblasts to apoptosis [59].

Despite the approval of pirfenidone [60] and nintedanib [61], IPF has a very poor prognosis with a life expectancy of 3–5 years once diagnosed [54]. Thus, lung transplantation still remains the only treatment option that markedly improves the quality of life and survival of IPF patients [50]. Both pirfenidone and nintedanib delay disease progression by exerting pleiotropic effects, which range from the inhibition of inflammatory processes to the blockage of fibroblast proliferation and ECM production. Although the pirfenidone mode of action remains elusive, several studies demonstrated the direct impact of this drug on the Hedgehog and TGF-β signaling pathways [60,62]. In contrast to pirfenidone, nintedanib is a tyrosine kinase inhibitor (TKI) of PDGF, VEGF, and fibroblast growth factor (FGF) receptors. In addition, it also inhibits a narrow range of other targets at pharmacologically-relevant doses including the Src family and Flt-3 kinases. Ligands of PDGF, VEGF, and FGF receptors are known to have potent profibrotic effects [61]. While the approval of pirfenidone and nintedanib was a milestone in the care of IPF, there is still a high and unmet clinical need in this patient group. A multi-targeted approach, potentially with combination therapies and the identification of subsets of IPF patients who may respond more favorably to specific agents, are likely to dominate future clinical studies. 

Targeting ErbB receptors and their ligands may serve as a potential therapeutic option for IPF, in particular, that different elements of the ErbB signaling can be pharmacologically targeted. However, a complex ligand–receptor network and the involvement of the ErbB signaling in the tissue regenerative process may encounter some unexpected surprises, thus further studies critically evaluating the role of ErbB receptors and their ligands in adaptive versus maladaptive remodeling of lung tissue are urgently needed.

## 3. ErbB Receptor–Ligand System in Lung Fibrosis

ErbB receptors and their ligands are expressed in a large variety of human tissues including the epithelial cells of the lung [63]. Under physiological conditions, ErbB1–4 are expressed in bronchial epithelial and alveolar type II (ATII) cells [64,65], whereas ErbB ligands, such as TGF-α, AREG, and HB-EGF, are expressed in bronchial epithelial cells [65]. Furthermore, TGF-α, AREG, HB-EGF, BTC, and EGF are produced in serous acinar cells from submucosal glands beneath the respiratory epithelium [64]. In cell culture, naïve lung fibroblasts were found to express TGF-α, HB-EGF, HRG and AREG but not BTC [66].

Increased expression of various ErbB ligands is associated with fibrosis development in multiple organs, including, lung, liver, or pancreas. For example, overexpression of HB-EGF and AREG causes pancreatic fibrosis [67,68], while high levels of AREG alone are sufficient to trigger liver fibrosis [69]. Furthermore, increased expression of EPG results in the fibrosis of the nerve system, and overexpression of TGF-α is associated with fibrosis of the lung [70]. Interestingly, deficiency of HB-EGF is linked to liver fibrosis [71], thus pointing towards a dual and organ-specific role of the ErbB receptor–ligand system in the tissue scarring processes. Below, we provide evidence for a dual (“good” versus “bad”) role of ErbB receptors and their ligands in lung fibrosis and, in particular, in IPF.

### 3.1. ErbB1/EGFR Receptor

Besides being overexpressed in many types of cancer, ErbB1 is also upregulated in lung epithelial cells from patients with different forms of pulmonary fibrosis [72]. In IPF, abundant ErbB1 immunostaining was found in the hyperplastic alveolar epithelium surrounding areas of fibrosis and inflammation. In addition, increased ErbB1 protein levels were reported in IPF fibroblastic foci and in fibroblasts isolated from IPF lungs [73]. Furthermore, IPF lung fibroblast (LF)-derived culture supernatants were found to stimulate expression of ErbB1 in donor LF in an FGF-dependent manner [73]. In addition, a negative correlation between ErbB1 mRNA levels and the indicators of IPF progression, such as forced vital capacity (FVC) and diffusion capacity of the lung for carbon monoxide (DLCO) [72], was reported. 

After the introduction of ErbB1 TKI to cancer therapy, the discussion on their repurposing and usage in the treatment of other hyperproliferative diseases, including lung fibrosis, began. Quickly, first studies demonstrated that tyrphostin AG1478 reduces proliferation of LF and attenuates pulmonary fibrosis caused by intratracheal instillation of vanadium pentoxide in rats [74]. Another ErbB1 TKI, gefitinib, suppressed proliferation of LF and diminished pulmonary fibrosis in the bleomycin-treated mice [75,76]. In contrast, Suzuki et al. [77] demonstrated that gefitinib aggravates bleomycin-induced lung fibrosis in mice by reducing the regenerative potential of alveolar epithelial cells. Although the reasons for these contradictory findings are unknown, differences in mouse strains, dosages, intervals and mode of drug application could have played a role. 

Development of acute lung injury and ILD in non-small cell lung cancer (NSCLC) patients receiving gefitinib demonstrates possible deleterious effects of the ErbB1 signaling inhibition [78,79]. Interestingly, similar harmful effects were also observed in NSCLC patients treated with another ErbB1 TKI, erlotinib [80,81]. The incidence of ILD in TKI-treated NSCLC patients is ~1% worldwide [82]. In the Japanese population, it is significantly higher at ~2%. Despite this observation, the *EGFR* polymorphism leading to the genetic susceptibility to the treatment with ErbB1 TKI in the Japanese was not observed [82]. Pre-existing lung disorders, such as interstitial pneumonia or pulmonary fibrosis, male sex and history of smoking, were identified as risk factors for the development of gefitinib-associated ILD [83,84]. Considering the chemical and pharmacological similarities between gefitinib and erlotinib, the same risk factors may apply to the erlotinib-trigger ILD. In addition, radio- and chemotherapy, both used to treat cancer, seem to aggravate ErbB1 TKI-induced ILD [85]. Currently, it is not known what mechanisms lead to the development of ILD in NSCLS patients receiving gefitinib or erlotinib. However, it is increasingly recognized that the border between adaptive and maladaptive repair of the lung tissue is thin and the clue to success is maintaining the balance between all the factors involved [86].

### 3.2. Transforming Growth Factor-α

Among all ErbB1 ligands, TGF-α is the one with a well-described function in pulmonary fibrosis. TGF-α was found to be overexpressed in ATII cells, endothelial cells and fibroblasts in the lungs of IPF patients [87]. In addition, its levels were reported to be increased in IPF bronchoalveolar lavage fluid (BALF) [88]. The profibrotic potential of TGF-α was demonstrated in several studies, in which lung-specific overexpression of TGF-α in mice was conducted. For example, chronic production of TGF-α in surfactant protein-C (SP-C)-expressing cells disrupted alveolar and vascular development and caused pulmonary fibrosis and pulmonary hypertension in mice [85]. Similarly, chronic conditional expression of TGF-α driven by the doxycycline-regulatable Clara cell secretory protein (dox-CCSP) promoter triggered progressive vascular adventitial, peribronchial, interstitial, and pleural fibrosis, which was independent of inflammation and TGF-β activation [89]. Further studies demonstrated transcriptional similarities between dox-CCSP-TGF-α-induced lung fibrosis and IPF, thus pointing towards an essential role of the ErbB1–TGF-α axis in the development of IPF. In the rat bleomycin model, increased immunoreactivity for TGF-α and ErbB1 was observed in macrophages, alveolar septal cells and respiratory epithelial cells. Both proteins were predominantly detected in foci of cellular proliferation and in areas of intra-alveolar fibrosis [90]. Accordingly, TGF-α-deficient mice showed reduced hydroxyproline levels and partially preserved lung structure following bleomycin application as compared to wild-type littermates [91]. Interestingly, overexpression of TGF-α under the control of SP-C promoter protected mice against acute lung injury was caused by inhalation of polytetrafluoroethylene (PTFE; teflon) fumes. Histological hallmarks of this model are pulmonary hemorrhage and inflammation. Indeed, SP-C-TGFα-transgenic mice exhibited reduced levels of IL-6 and macrophage inflammatory protein 2 in lung homogenates and decreased total protein levels and neutrophil numbers in BALF as compared to non-transgenic controls. Altogether, these findings demonstrate the etiology-dependent role of TGF-α in lung pathologies. 

In line with these observations, ErbB1 TKI, gefitinib, partially reduced collagen levels and improved lung compliance, tissue and airway elastance, and airway resistance in mice overexpressing TGF-α under tetracycline-inducible CCSP (rtTA-CCSP) promoter [89]. These changes were supported by the decreased expression of several genes associated with lung parenchymal and vascular remodeling. It is worth mentioning here that gefitinib neither induced chronic lung injury nor exacerbated pulmonary fibrosis, thus supporting further studies to determine the role of ErbB1 in human lung fibrotic diseases [85,92]. Furthermore, the same group demonstrated that blockage of the ErbB1 downstream signaling mediator, PI3K, by the PX-866 pan-inhibitor reduced total lung collagen content and improved pulmonary mechanics in rtTA-CCSP-TGF-α overexpressing mice [93]. These results were recapitulated when another ErbB1 signaling pathway, MAPK/ERK, was targeted. Administration of an allosteric MEK inhibitor, ARRY-142886, prevented the progression of established lung fibrosis in rtTA-CCSP-TGF-α overexpressing mice [94]. To sum up, a growing body of evidence suggests that TGF-α-driven activation of the ErbB1 signaling pathways may play an important role in the development of lung fibrosis and that TGF-α might be amenable to targeted therapy.

### 3.3. Amphiregulin

Amphiregulin, another ErbB1 ligand, was discussed in the context of maladaptive remodeling of the liver and lung. In this respect, AREG-deficient mice were found to be protected against liver fibrosis induced by chronic administration of carbon tetrachloride (CCl_4_) [69]. To decipher the underlying molecular mechanism, several studies focused on the link between AREG and a master regulator of fibrogenesis, TGF-β1. Zhou et al. [95] reported that stimulation of fibroblasts with TGF-β1 elevates AREG production, which in turn increases cell proliferation and the expression of profibrotic genes, such as α-smooth muscle actin, collagen 1-α1/α2, fibronectin and tenascin. These effects were reversed by the treatment of TGF-β1-stimulated fibroblasts with AREG siRNA or ErbB1 inhibitors, AG1478 or gefitinib [95]. Consistent with these in vitro findings, AREG expression was markedly increased in the lungs of dox-CC10-TGF-β1 overexpressing mice and administration of AREG siRNA or AG1478 reduced collagen content and attenuated lung fibrosis in these animals. Besides AREG, the increased expression of other ErbB1 ligands, such as EREG and HB-EGF following exposure of fibroblasts to TGF-β1, was reported [96,97]. Andrianifahanana et al. [97] documented that TGFβ-induced AREG, EREG, and HB-EGF production requires the integration of an autocrine signal from a PDGF receptor and engages a positive feedback loop through ErbB1. The same authors demonstrated the pathological relevance of PDGFR-ErbB1 cooperation in the bleomycin model of lung fibrosis. Namely, they observed that simultaneous application of imatinib (a PDGF receptor inhibitor) and lapatinib (an ErbB1/2 inhibitor) is more effective than either treatment alone. Although, there is no evidence that pirfenidone and nintedanib directly interfere with the ErbB1, their ability to inhibit TGFβ and VEGF/PDGF/FGF receptors, respectively, might influence the overall ErbB1 activity in lung fibrosis. Accordingly, Shochet et al. reported that ErbB1 expression in donor LF triggered by IPF LF-culture media depends on FGF and can be reversed by nintedanib [73]. Interconnections of ErbB1 with other signaling pathways have to be considered when designing future IPF therapies. 

The complexity of AREG cellular effects is underscored by the recent publication by Stancil et al., who showed the role of the AREG–ErbB1 axis in the jamming–unjamming of airway epithelial cells in IPF. Jamming transition describes a process of epithelial cell transformation from migratory (unjammed) to non-migratory (jammed) status in the absence of wounding or cell-type changes. This transition is believed to play an important role during embryogenesis, in processes such as axis elongation and tissue development [98,99,100]. It was also associated with the pathogenesis of carcinomas [101] and asthma [102]. Stancil et al. [103] demonstrated in vitro that the unjammed phase is extended in distal airway epithelial cells of IPF patients and is associated with increased activity of the ErbB-YAP (Yes-Associated Protein) signaling pathway. YAP is a transcriptional co-activator, which was found to regulate epithelial progenitor cell proliferation in the lung [104] and epithelial–mesenchymal transition in lung cancer cells following exposure to TGF-β [105]. These findings are supported by the increased levels of AREG in IPF distal airway epithelial cells and its ability to induce jammed to unjammed transition in controlling distal airway epithelial cells in vitro [103]. Interestingly, the AREG-triggered extended unjammed phase of distal airway epithelial cells correlated with activation of the fibroblasts lying underneath [103], thus providing ample evidence for the contribution of airways epithelial cells repopulating distal parts of IPF lungs to the disease progression. The association between the AREG-driven prolonged unjammed status of distal airway epithelial cells and the gain-of-function *MUC5B* promotor variant underscores this assumption. 

Besides its “bad” role in lung fibrosis, AREG was also found to contribute to the restoration of tissue homeostasis after acute lung injury driven by infection. Minutti et al. [106] showed that macrophage-derived AREG promotes TGF-β1 activation and subsequent differentiation of pericytes into collagen-producing myofibroblasts leading to restoration of vascular integrity in injured tissue and wound healing. Thus, not only TGF-β1 may regulate AREG expression but vice versa AREG can control the levels of active TGF-β1. It seems that the first scenario operates in fibrosis and the second under inflammatory conditions. Thus, the function of AREG may depend on its source, concentration and cellular and molecular landscape of the surrounding area. Although further research is needed to decipher the function of AREG in acute versus chronic pathological conditions, it becomes clear that identification of dynamics and causal flows in complex AREG signaling networks is crucial for its use as a therapeutic target. This assumption is supported by the study demonstrating attenuation of bleomycin-induced lung fibrosis upon AREG application during the late inflammatory phase [107]. This observation is in sharp contrast to the lung fibrosis reports mentioned above but it may be explained by the findings of Minutti et al. [106], namely, the AREG effects on blood vessel regeneration and thus epithelial cell survival in acute lung injury [95]. Overall, it seems that AREG properties may depend on the genetic background and the immune system condition, thus preselecting the potential responders prior to the treatment may raise the possibility of the success of an anti-AREG therapy in IPF.

### 3.4. ErbB2 and ErbB3 Receptors and Their Ligands

Another ErbB receptor that was linked to pulmonary fibrosis is ErbB2. Besides being an important oncogene in breast and ovarian cancer [44], ErbB2 was found to be involved in epithelial cell recovery upon acute lung injury. While ErbB2 was detected on the basolateral side of airway epithelial cells, HRG-α was only found in the apical membrane of these cells and in the overlying mucus film [108]. When epithelial integrity is disrupted, HRG-α translocates to Erb2 and enables a rapid response to injury. Thus, the Erb2-HRG-α systems sense changes in the extracellular environment and ensure restoration of barrier function that may be critical for survival. As there is no known ligand for ErbB2, this receptor is able to transduce intracellular signaling only upon forming a complex with other ErbB receptors. In pulmonary epithelial cells, ErbB2 is the preferred binding partner for ErbB3. Besides being engaged in the HRG-α-triggered intracellular signaling, the ErbB2–ErbB3 complex also responds to NRG1. Using a dominant-negative mutant of ErbB3 expressed under the SP-C promotor, Nethery et al. [109] demonstrated that SP-C-ErbB3 transgenic mice exhibit reduce collagen levels in the lung and better survival following bleomycin administration. The effect was associated with the inability of NRG1 to signal *via* the nonfunctional ErbB2–ErbB3 complex. These findings are corroborated by Faress et al. [110], who reported preserved lung structure and diminished lung collagen content upon administration of an anti-ErbB2 antibody (2C4) to bleomycin-treated mice.

The important role of ErbB2 in bronchial epithelial cell differentiation and proliferation was shown by Vermeer et al. [66]. These authors demonstrated that treatment of airway epithelial cells with an anti-ErbB2 antibody, trastuzumab, induces their de-differentiation associated with an increase in the numbers of non-ciliated and metaplastic, flat cells. By contrast, the exposure of the cells to HRG-α preserved normal differentiation of airway epithelial cells. Most interestingly, co-culturing of airway epithelial cells with fibroblasts potentiated epithelial cell differentiation comparable to that achieved following treatment with HRG-α, pointing towards the ability of fibroblasts to produce ErbB ligands. Indeed, further studies demonstrated that normal human LF express TGF-α, HB-EGF, EREG, AREG, and HRG-α [66]. These observations were in line with the clinical case report describing reversible changes in airway epithelial cell differentiation of a breast cancer patient that coincided with the initiation and discontinuation of a trastuzumab therapy [66].

The ErbB3–NRG1-α axis was also discussed in the context of alveolar bronchiolization seen in the lungs of IPF patients. In these patients, NRG1-α was detected in epithelial cells lining honeycombing areas, as well as in normal submucosal glands [111]. In addition, elevated levels of this molecule were measured in IPF BALF [112]. Given the ability of NRG1-α to regulate airway mucus cell differentiation and MUC5B expression, it is worth speculating about its pivotal role in airway epithelial cell reprogramming and thus honeycomb cyst formation in IPF [113]. 

Taken together, it seems that ErbB2–ErbB3 activation is essential for the differentiation of airway epithelial cells and their integrity. Overactivation of this receptor complex system may induce abnormal behavior of airway epithelial cells thereby contributing to the honeycomb cyst formation and fibrosis progression. Because of the risks associated with the ErbB2–ErbB3 complex inhibition, close patient monitoring and patient categorization have to be taken into account when considering an anti-ErbB2–ErbB3 therapy in IPF.

## 4. Conclusions

**Figure 2:**
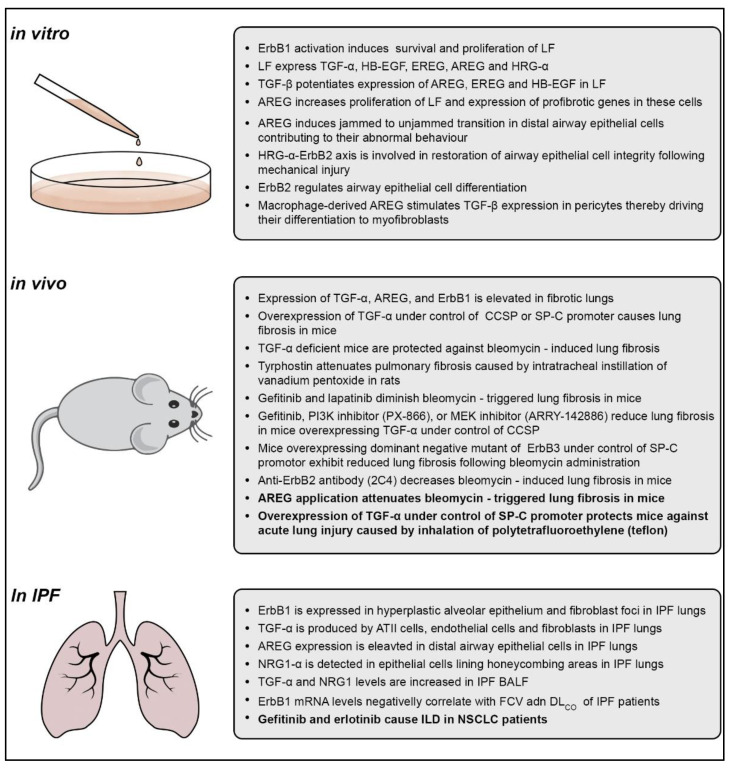
**Overview of the in vitro, in vivo and clinical findings for the role of the ErbB/ligand system in lung fibrosis.** LF, lung fibroblasts; TGFα, transforming growth factor-α; HB-EGF, heparin-binding epidermal growth factor-like growth factor; EREG, epiregulin; ***AREG***, amphiregulin; HRG-α, heregulins; TGF-β, transforming growth factor-β; CCSP, Clara cell secretory protein; SP-C, surfactant protein-C; PI3K, phosphoinositide 3-kinase; MEK, mitogen-activated protein kinase kinase; IPF, idiopathic pulmonary fibrosis; ATII cells, alveolar type II cells; NRG1α, neuregulin-1α; BALF, bronchoalveolar lavage fluid; FVC, forced vital capacity; DL_CO_, diffusion capacity of the lung for carbon monoxide; ILD, interstitial lung disease; NSCLC, non-small-cell lung cancer.

A growing body of evidence suggests the pivotal role of the ErbB-ligand system in irreversible lung tissue scarring (Figure 2). The ErbB receptors and ligands were found to be overexpressed in IPF lungs and a number of preclinical studies demonstrated their pro-fibrotic properties in the loss-of-function and gain-of-function approaches. In addition, the therapeutic application of ErbB receptor/ligand inhibitors was often associated with favorable outcomes in lung fibrosis models (see Table 1). However, in view of the multifunctionality of the ErbB receptor–ligand system and its role in tissue regeneration, concern remains. Identification of dynamics and causal flows in the ligand–ErbB signaling network in acute versus chronic lung injury will be a prerequisite to maximize the chance of success of anti-ErbB/ligand agents in the clinical trials for IPF.

In addition, a lesson has to be also drawn from the remarkable progress in understanding the ErbB biology in cancer. It is now clear that the results of clinical trials can only be improved by taking into account a number of important issues. First, the effects of the targeted therapy may be weakened because of differences in etiology and heterogeneity. Furthermore, stratification of the patients according to a predominant disease mechanism have to be considered. Molecular endotyping should be integrated into the protocols of clinical trials. This strategy may promote the prudent use of novel targeted therapies. Finally, identification of the factors that can predict drug response or resistance will play a fundamental role to tailor individual ErbB-based therapy regimens.

## Figures and Tables

**Table 1:** Anti-ErbB/ligand approaches in preclinical models of lung fibrosis.

Targeted Molecule	Inhibitor/ Antibody	Animal Model	Outcome	Reference
ErbB1	AG1478	Vanadium pentoxide	Favorable	Rice et al., 1999 [73]
ErbB1	Gefitinib	Bleomycin	Favorable	Ishii et al., 2006 [75]; Wang et al., 2010 [74]
ErbB1	Gefitinib	Bleomycin	Harmful	Suzuki et al., 2003 [76]
ErbB1	Gefitinib	rtTA-CCSP ^4^-TGF-α ^5^	Favorable	Hardie et al. 2004 [88]
PI3K ^1^	PX-866	rtTA-CCSP-TGF-α	Favorable	Hardie et al., 2010 [92]
MEK ^2^	ARRY-142886	rtTA-CCSP-TGF-α	Favorable	Madala et al., 2012 [93]
AREG ^3^	AREG siRNA	dox-CC10 ^6^-TGF-β1 ^7^	Favorable	Zhou et al., 2012 [94]
ErbB1	AG1478	dox-CC10-TGF-β1	Favorable	Zhou et al., 2012 [94]
ErbB1/2	Lapatinib	Bleomycin	Favorable	Andrianifahanana et al., 2013 [96]
ErbB2	anti-ErbB2 antibody (2C4)	Bleomycin	Favorable	Faress et al., 2007 [109]

^1^ PI3K, phosphoinositide 3-kinase; ^2^ MEK, mitogen-activated protein kinase; ^3^ AREG, amphiregulin; ^4^ rtTA-CCSP, tetracycline-inducible Clara cell secretory protein; ^5^ TGF-α, transforming growth factor α; ^6^ dox-CC10, doxycycline-regulatable Clara cell 10-kDa protein; ^7^ TGF-β, transforming growth factor β1.

## Data Availability

Not applicable.
